# Comment on “Preemptive left stellate ganglion block reduces the incidence and severity of cardiac surgery-associated acute kidney injury: a randomized clinical trial”

**DOI:** 10.1097/JS9.0000000000003204

**Published:** 2025-08-23

**Authors:** Xinni Ye, Haifeng Wang, Shi Fu

**Affiliations:** Department of Urology, The Second Affiliated Hospital of Kunming Medical University, Yunnan, China

*Dear Editor,*

We read with great interest the randomized clinical trial by Zhou *et al*^[1]^, which demonstrated that preemptive left stellate ganglion block (SGB) significantly reduced the incidence and severity of cardiac surgery-associated acute kidney injury (CSA-AKI) in patients undergoing cardiopulmonary bypass (CPB), while improving renal perfusion and systemic inflammatory markers. This study proposed a novel neuro-immune-renal perfusion intervention pathway and highlighted perioperative autonomic modulation as a promising kidney-protective strategy, deserving further investigation in perioperative organ protection. However, we believe there are several aspects that could be further refined. In preparing this commentary, we ensured compliance with the TITAN 2025 guidelines for responsible AI use in academic communication^[2]^.

According to the joint guidelines by the Society of Thoracic Surgeons, the Society of Cardiovascular Anesthesiologists, and the American Society of ExtraCorporeal Technology, the diagnosis of AKI after cardiac surgery should be based on a combination of serum creatinine and urine output^[3]^. Serum creatinine has limited sensitivity to changes in glomerular filtration rate and is delayed in reflecting kidney injury. In non-steady-state conditions such as post-cardiac surgery, creatinine may be diluted by fluid overload, leading to delayed diagnosis and underestimation of renal injury^[4]^. In perioperative patients receiving CPB and diuretics, urine output may be affected. However, it remains a valuable indicator of renal perfusion trends and enhances the sensitivity of AKI detection. Especially during postoperative recovery, sustained oliguria or anuria may indicate poor prognosis and should receive clinical attention^[5]^.

To overcome the limitations of serum creatinine and urine output, recent studies have focused on the potential of urinary biomarkers for early detection and risk stratification. For example, IL-18 and kidney injury molecule-1 increase before serum creatinine rises, indicating subclinical AKI and providing prognostic value for mortality. Thus, developing and applying such biomarkers may allow earlier identification and improved prediction of CSA-AKI^[6]^.

Regarding covariate control, preoperative systemic comorbidities such as chronic lung disease and perioperative anemia were not adjusted for in the randomization or regression models. In addition, postoperative management – including fluid strategy, vasopressor use, and timing of renal replacement therapy – may have impacted outcomes but lacked systematic documentation and comparison^[6,7]^.

Although variables such as sex, age, cardiac function, hypertension, diabetes, and surgical data were included, no subgroup analysis based on preoperative or intraoperative risk factors was conducted. Thus, the protective effect of left SGB across varying risk strata remains unclear. Recent studies have developed predictive models incorporating preoperative and intraoperative variables, with nomograms being particularly useful for individualized CSA-AKI risk assessment before surgery^[8]^. Incorporating intervention variables into these models and analyzing effects by risk strata, may enhance clinical relevance and reveal the precision impact of interventions in specific populations.

Finally, this was a single-center study with a small sample size and strict exclusion of high-risk patients, such as those with renal insufficiency, arrhythmia, repeat surgery, or aortic procedures, limiting generalizability. In real-world settings, perioperative risk assessment and management vary across hospitals, suggesting that selection bias may limit the representativeness of the study population^[9,10]^.

In conclusion, Zhou *et al* have presented a pioneering study that advances the clinical translation of perioperative neuromodulation for renal protection. We look forward to future studies with larger samples, multi-center designs, and long-term follow-up, integrating early biomarkers and predictive models to validate the clinical utility and generalizability of SGB in the prevention and treatment of CSA-AKI.

## Data Availability

Not applicable.
